# Supplementary material for: Can a specific biobehavioral-based therapeutic education program lead to changes in pain perception and brain plasticity biomarkers in chronic pain patients? A study protocol for a randomized clinical trial
Source: PLoS One. 2024 Jan 19;19(1):e0289430. doi: 10.1371/journal.pone.0289430 (PMC10798500; doi:10.1371/journal.pone.0289430)
Supplement: S1 Checklist — (DOCX) [file pone.0289430.s001.docx]

***Annex1. TiDier checklist for intervention group***

| **CATEGORY** | **ITEM N.:** | **DESCRIPTION** |
| --- | --- | --- |
| **BRIEF NAME** | 1 | Provide the name or a phrase that describes the intervention.  *Pain Oriented Biobehavioral Therapeutic Education (POBTE) + Exercise* |
| **WHY** | 2 | Describe any rationale, theory, or goal of the elements essential to the intervention*:*   *To study the effects of TE to reduce perceived pain intensity and increase BDNF levels in patients with chronic pain.* |
| **WHAT** | 3 | Materials: Describe any physical or informational materials used in the intervention, including those provided to participants or used in intervention delivery or in training of intervention providers. Provide information on where the materials can be accessed (e.g. online appendix, URL*)*  *The patients assigned to this group will be guided by a physiotherapist who will use his material that he will make available to the patients (power point presentation, notebook, explanatory self-created video about pain).* |
|  | 4 | Procedures: Describe each of the procedures, activities, and/or processes used in the intervention, including any enabling or support activities.  *Session 1: Presentation, motivational interview, therapeutic contract.*  *Session 2: Reconceptualization of pain (1st part): damage and pain, subjectivity. Motor imagery (Observed Actions + Explicit Imagery).*  *Session 3: First part concepts comprehension test and recall. Reconceptualization of pain (2nd part): neuroplasticity, homunculus. Beliefs + expectations (influence on pain perception, coping behavior...).*  *Session 4: Self-management of sensory aspects (coping strategies, recovery times...). Recall new concepts (1st and 2nd part).*  *Session 5: General treatment feedback according to the profile. Reconceptualization of pain (3rd part): influence of the context, attention, etc...*  *Session 6: Recall new concepts 1st to 3rd part and test. Motor imagery dosage test and strengthen concept. Recall of beliefs and expectations.*  *Session 7: Stress management skills and sleep disturbances on pain. Setting future goals and objectives. Self-monitoring (Review of pain diaries...). Reconceptualization of pain (4th part): relapses, social support....*  *Session 8: Global assessment of education (ad hoc concepts exam). Individualised take home messages and feedback on the evolution.* |
| **WHO PROVIDED** | 5 | For each category of intervention provider (e.g. psychologist, nursing assistant), describe their expertise, background and any specific training given*.*    For the therapeutic education sessions, all educators will be healthcare professionals (physiotherapists) with at least 15 years of experience with this educational model and patients with persistent pain. |
| **HOW** | 6 | Describe the modes of delivery (e.g. face-to-face or by some other mechanism, such as internet or telephone) of the intervention and whether it was provided individually or in a group*:*     *Face to face, small group education (5/6 people).* |
| **WHERE** | 7 | Describe the type(s) of location(s) where the intervention occurred, including any necessary infrastructure or relevant features*:*  *Rey Juan Carlos University, Alcorcón (Madrid). Spain* |
| **WHEN and HOW MUCH** | 8 | Describe the number of times the intervention was delivered and over what period of time including the number of sessions, their schedule, and their duration, intensity or dose.   *POBTE: 2/week, maximum 45 minutes each session. 4 weeks.*  *Exercise: 3/week, maximum 45 minutes each session. 4 weeks.* |
| **TAILORING** | 9 | If the intervention was planned to be personalised, titrated or adapted, then describe what, why, when, and how*:*    */* |
| **MODIFICATIONS** | 10 | If the intervention was modified during the course of the study, describe the changes (what, why, when, and how*):*    *Everyone will start with little or no knowledge of the neuroscience of pain and progress from there with increasingly difficult classes.* |
| **HOW WELL** | 11 | Planned: If intervention adherence or fidelity was assessed, describe how and by whom, and if any strategies were used to maintain or improve fidelity, describe them*:*    *Adherence to supervised sessions and completion of the pain diary & ad hoc tests will be recorded as attendance.* |
|  | 12 | Actual: If intervention adherence or fidelity was assessed, describe the extent to which the intervention was delivered as planned.    *Adherence to supervised sessions will be recorded as attendance.* |
