# Supplementary material for: Can a specific biobehavioral-based therapeutic education program lead to changes in pain perception and brain plasticity biomarkers in chronic pain patients? A study protocol for a randomized clinical trial
Source: PLoS One. 2024 Jan 19;19(1):e0289430. doi: 10.1371/journal.pone.0289430 (PMC10798500; doi:10.1371/journal.pone.0289430)
Supplement: S2 Checklist — (DOCX) [file pone.0289430.s002.docx]

***Annex 2. CERT checklist for both groups***

| **CATEGORY** | **ITEM N.:** | **DESCRIPTION** |
| --- | --- | --- |
| **WHAT: materials** | 1 | Detailed description of the type of exercise equipment (e.g. weights, exercise equipment such as machines, treadmill, bicycle ergometer etc):    *Cycloergometer aerobic exercise* |
| **WHO: provider** | 2 | Detailed description of the qualifications, teaching/supervising expertise, and/or training undertaken by the exercise instructor:    *Physiotherapist (health professional)* |
| **HOW: delivery** | 3 | Describe whether exercises are performed individually or in a group:    *In a group, on individual machines* |
|  | 4 | Describe whether exercises are supervised or unsupervised and how they are delivered:    *Supervised* |
|  | 5 | Detailed description of how adherence to exercise is measured and reported:    *Adherence to supervised sessions will be recorded as attendance.* |
|  | 6 | Detailed description of motivation strategies:    *Ongoing communication with positive reinforcement from the healthcare provider.* |
|  | 7a | Detailed description of the decision rule(s) for determining exercise progression:    *Functional capacity (heart rate* |
|  | 7b | Detailed description of how the exercise program was progressed:    *Functional capacity (heart rate)* |
|  | 8 | Detailed description of each exercise to enable replication (e.g. photographs, illustrations , video etc):    *Practical explanation* |
|  | 9 | Detailed description of any home program component (e.g. other exercises, stretching etc)    *Warm up and cool down with mobility exercises* |
|  | 10 | Describe whether there are any non-exercise components (e.g. education, cognitive behavioural therapy, massage etc):    *Therapeutic education (POBTE method) in the intervention group.* |
|  | 11 | Describe the type and number of adverse events that occurred during exercise:    *Musculoskeletal injury. Recording and interrupting the trial.* |
| **WHERE: location** | 12 | Describe the setting in which the exercises are performed:    *Rey Juan Carlos University, Alcorcón (Madrid), Spain.* |
| **WHEN, HOW MUCH: dosage** | 13 | Detailed description of the exercise intervention including, but not limited to, number of exercise repetitions/sets/sessions, session duration, intervention/program duration etc:    *3 sessions/week of a maximum of 45 minutes per session for 4 weeks. Intensity according to personal heart rate.* |
| **TAILORING: what, how** | 14a | Describe whether the exercises are generic (one size fits all) or tailored whether tailored to the individual:      *Individualized according to heart rate* |
|  | 14b | Detailed description of how exercises are tailored to the individual    *Heart rate measured with pulsi oximeter* |
|  | 15 | Describe the decision rule for determining the starting level at which people commence an exercise program (such as beginner, intermediate, advanced etc):  *Heart Rate* |
| **HOW WELL: planned, actual** | 16a | Describe how adherence or fidelity to the exercise intervention is assessed/measured:    *Joining sessions will be recorded as adherence (at least 80%).* |
|  | 16b | Describe the extent to which the intervention was delivered as planned      / |
