# Supplementary material for: Can a specific biobehavioral-based therapeutic education program lead to changes in pain perception and brain plasticity biomarkers in chronic pain patients? A study protocol for a randomized clinical trial
Source: PLoS One. 2024 Jan 19;19(1):e0289430. doi: 10.1371/journal.pone.0289430 (PMC10798500; doi:10.1371/journal.pone.0289430)
Supplement: S3 Checklist — (DOCX) [file pone.0289430.s003.docx]

| **BRIEF NAME** |
| --- |
| **1. INTERVENTION: Provide a brief description of the educational intervention for all groups involved [e.g. control and comparator(s)].**  Aerobic exercise with cycloergometer will be performed in both the control group and the intervention group. In the latter, in addition, eight sessions of Physiotherapy-Oriented Behavioural Therapeutic Education will be carried out. |
| **WHY - this educational process** |
| **2. THEORY: Describe the educational theory (ies), concept or approach used in the intervention.**  POBTE (Physiotherapy-Oriented Behavioural Therapeutic Education) aims to address this gap by implementing a proven model used in promoting health-related behaviors, focusing on cultivating new behaviors and decision-making skills. The intervention draws on the meaningful learning model, incorporating cognitive concepts related to pain processing and salutogenic principles to improve pain understanding and overall well-being. It also utilizes modified Bloom's taxonomy to prioritize lower-order thoughts initially and applies the flipped classroom model to bridge existing and future content, considering the neurocognitive changes associated with chronic pain. Reinforcement content, activities in personal spaces, and adaptations based on retention rates and consolidation times are integrated, while motivational interviewing processes and biobehavioral strategies such as therapeutic contracts and movement representation are incorporated to enhance adherence and coping capacities. |
| **3. LEARNING OBJECTIVES: Describe the learning objectives for all groups involved in the educational intervention.**  The POBTE model provides a holistic and patient-centered approach that aims to induce meaningful behavioral changes and improve decision-making skills regarding pain management. |
| **4. EBP CONTENT: List the foundation steps of EBP (ask, acquire, appraise, apply, assess) included in the educational intervention.**  The step of this educational model with follow the Meaningful Learning Based Design (motivation, activation, new information entry, comprehension, learning by doing, repetition, challenge, diagnostics) and the six Bloom’s Taxonomy levels (remembering, understanding, applying, analyzing, evaluating, and creating). |
| **WHAT** |
| **5. MATERIALS: Describe the specific educational materials used in the educational intervention. Include materials provided to the learners and those used in the training of educational intervention providers**  During the education classes we will make use of support material such as power point and explanatory videos. At the end of the class, written notes will be provided to the patients so that they can review and revise them at home as often as they wish. Finally, at the end of each learning block, there will be an ad hoc test to assess knowledge. |
| **6. EDUCATIONAL STRATEGIES: Describe the teaching/learning strategies (e.g. tutorials, lectures, online modules) used in the educational intervention.**  Within the educational strategies explained above, the active participation of the patient in class will be encouraged and the lecturer will be available to resolve any doubts that may arise outside the class. |
| **7. INCENTIVES: Describe any incentives or reimbursements provided to the learners.**  Due to the policy of our ethics committee, no financial resources will be provided to patients (e.g. travel assistance). However, free educational resources such as course notes will be included. |
| **WHO PROVIDED** |
| **8. INSTRUCTORS: For each instructor(s) involved in the educational intervention describe their professional discipline, teaching experience/expertise. Include any specific training related to the educational intervention provided for the instructor(s).**  For the therapeutic education sessions, all educators will be healthcare professionals (physiotherapists) with at least 15 years of experience with this educational model and patients with persistent pain. |
| **HOW** |
| **9. DELIVERY: Describe the modes of delivery (e.g. face-to-face, internet or independent study package) of the educational intervention. Include whether the intervention was provided individually or in a group and the ratio of learners to instructors.**  Education will be conducted in small groups (maximum 5 or 6 people), in order to maximise the benefits of group learning with the possible individualisation due to the small group. In addition, we want to take advantage of the benefits of face-to-face learning and encourage the therapeutic alliance with the professional and therefore, it will be face-to-face. |
| **WHERE** |
| **10. ENVIRONMENT: Describe the relevant physical learning spaces (e.g. conference, university lecture theatre, hospital ward, community) where the teaching/learning occurred.**  All sessions will be conducted in a designated university classroom, featuring an oval seating arrangement to promote communication among participants. Additionally, a screen will be available to project educational content, ensuring clear visibility for everyone. |
| **WHEN and HOW MUCH** |
| **11. SCHEDULE: Describe the scheduling of the educational intervention including the number of sessions, their frequency, timing and duration.**  *Number of sessions:* 8.  *Frequency:* 2 times a week.  *Duration:* 45 minutes.  *Time:* 6pm-6.45pm.  **Schedule:**  Session 1: Presentation, motivational interview, therapeutic contract.  Session 2: Reconceptualization of pain (1st part): damage and pain, subjectivity. Motor imagery (Observed Actions + Explicit Imagery).  Session 3: First part concepts comprehension test and recall. Reconceptualization of pain (2nd part): neuroplasticity, homunculus. Beliefs + expectations (influence on pain perception, coping behavior...).  Session 4: Self-management of sensory aspects (coping strategies, recovery times...). Recall new concepts (1st and 2nd part).  Session 5: General treatment feedback according to the profile. Reconceptualization of pain (3rd part): influence of the context, attention, etc...  Session 6: Recall new concepts 1st to 3rd part and test. Motor imagery dosage test and strengthen concept. Recall of beliefs and expectations.  Session 7: Stress management skills and sleep disturbances on pain. Setting future goals and objectives. Self-monitoring (Review of pain diaries...). Reconceptualization of pain (4th part): relapses, social support....  Session 8: Global assessment of education (ad hoc concepts exam). Individualised take home messages and feedback on the evolution. |
| **12. Describe the amount of time learners spent in face to face contact with instructors and any designated time spent in self-directed learning activities.**  Face-to-face education will require 45 minutes twice a week, while individual learning at home may vary from patient to patient. However, patients will be asked to dedicate at least 15 minutes a day to review the concepts seen in class in order to consolidate their learning. |
| **PLANNED CHANGES** |
| **13. Did the educational intervention require specific adaptation for the learners? If yes, please describe the adaptations made for the learner(s) or group(s).**  No specific adaptations are required. |
| **UNPLANNED CHANGES** |
| **14. Was the educational intervention modified during the course of the study? If yes, describe the changes (what, why, when, and how).**  Having tested the educational model with a large number of patients, we believe that now it fits the learning times of people in pain. However, some patients may need more time and/or concepts clarification between sessions. For this, the educators will be available to answer questions every day before and after the class, but no specific adaptation is required. |
| **HOW WELL** |
| **15. ATTENDANCE: Describe the learner attendance, including how this was assessed and by whom. Describe any strategies that were used to facilitate attendance.**  A record of participants' attendance will be kept. In addition, to encourage attendance, reminders will be sent out the day before the session. |
| **16. Describe any processes used to determine whether the materials (item 5) and the educational strategies (item 6) used in the educational intervention were delivered as originally planned.**  No changes are planned. |
| **17. Describe the extent to which the number of sessions, their frequency, timing and duration for the educational intervention were delivered as scheduled (item 11).**  Eight therapeutic education sessions (twice a week for 4 weeks) will be carried out for a maximum duration of 45 minutes as this has been found to be the optimal duration for patients with chronic pain. |
