# Supplementary material for: Can a specific biobehavioral-based therapeutic education program lead to changes in pain perception and brain plasticity biomarkers in chronic pain patients? A study protocol for a randomized clinical trial
Source: PLoS One. 2024 Jan 19;19(1):e0289430. doi: 10.1371/journal.pone.0289430 (PMC10798500; doi:10.1371/journal.pone.0289430)
Supplement: S1 File — (DOCX) [file pone.0289430.s005.docx]

***Conceptual Framework for Intervention with POBTE***

A major concern within the existing literature on educational strategies for the relief of persistent pain is the short-sighted focus on improving patient's understanding of the physiological processes that are involved in pain. This approach, while important, neglects key aspects essential to effectuating substantive changes in pain-related behavior [73]. As posited by Sullivan [74]: *individuals with pain differ from individuals without pain not only in how they “feel” but they differ in how they “behave”*. With this principle in mind, there is a need for a conceptual framework aimed at inducing behavioral changes from a physiotherapy perspective, rather than simply reducing the intensity or duration of pain. As such we have chosen to implement a proven model, previously used to improve health-related behaviors such as smoking cessation, dietary improvement, or physical activity promotion, as the basic structure for the POBTE approach.

This model seeks to cultivate the ability to establish new behaviors and to foster improved decision-making skills that facilitate the extrapolation of learned insights to different scenarios. It considers both environmental factors - encompassing the social and immediate environment - and personal factors, which relate to cognitive (what one knows), emotional (what one feels and believes) and skill (what one can do) domains, as schematically illustrated in Figure 6.


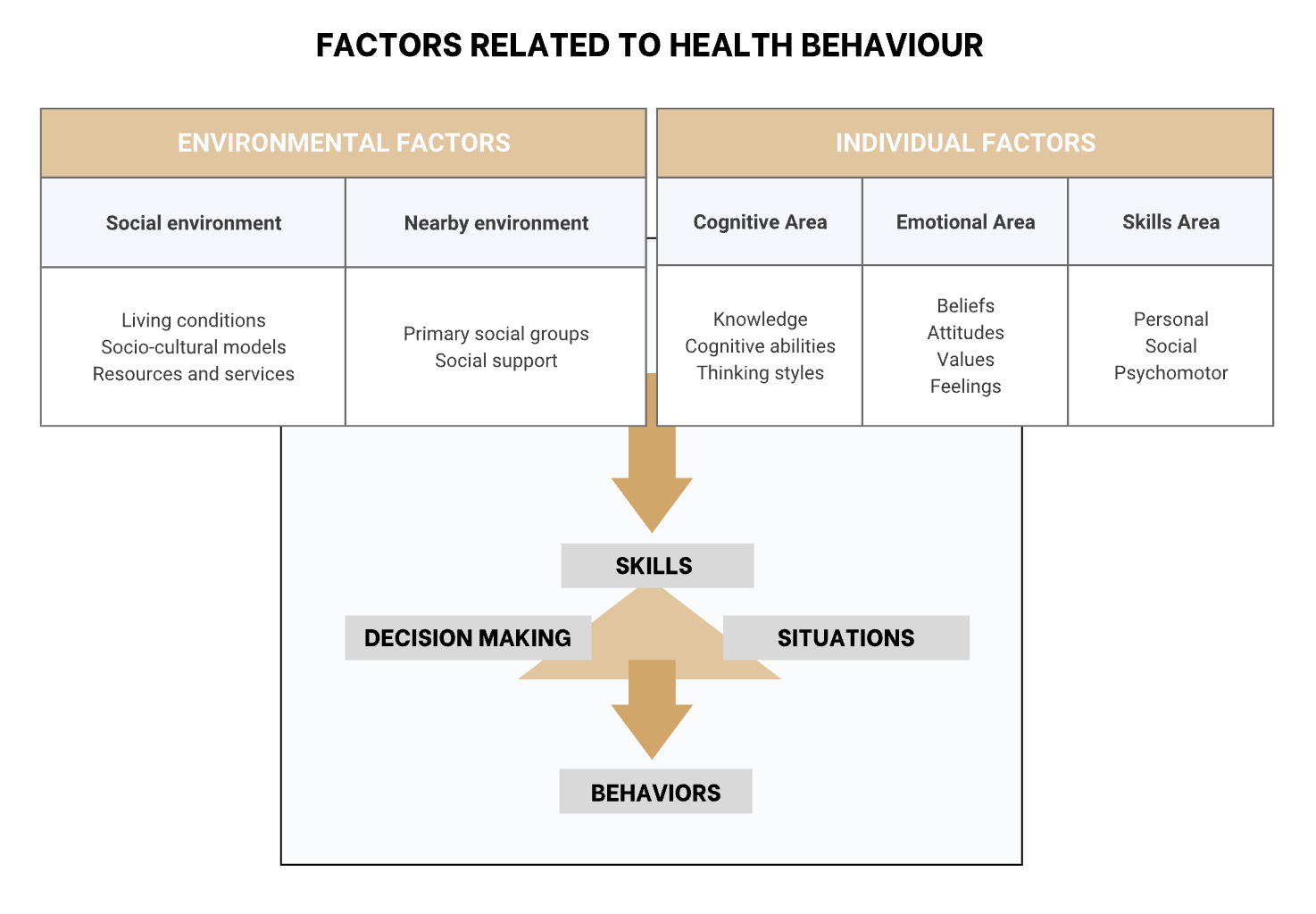


Figure 6. Adapted from*: “Manual de educación para la salud” Pérez-Jarauta, MC, 2006. Gobierno de Navarra. Spain*

Based on content developed in numerous interventions as reported in Pain Neuroscience Education articles [16], which incorporate cognitive domain concepts such as differentiating between damage and pain, tissue healing times, neuroplasticity, efferent copying, etc., as detailed in Supporting Material File 1 and 3 via the TiDier and GREET checklist, the intervention was designed to promote meaningful learning for patients from a salutogenesis perspective, with the goal of improving pain understanding and overall well-being [75].

For this intervention, Ausbel's model [76] of meaningful learning has been updated to reflect advances in learning neuroscience over the past 20 years [77], but retains the central tenet that *"the most important single factor influencing learning is what the learner already knows. Find out and teach accordingly"* [78]. Therefore, to catalyze a true reconceptualization of pain in patients, it is imperative to establish an initial base of what patients know, their previous motivations, and to progressively build the new concept on the foundations of current knowledge of pain processing.

In alignment with the salutogenic perspective mentioned, the POBTE approach's proposed content and activities aim to cultivate a "*Sense of Coherence*" among participants within the health-illness continuum, adhering to Antonovski's recommendation [79] to identify patients' "*general resistance resources*" within their unique contexts, with a view to transforming these into health assets, thereby fostering the development of necessary coping capacities to confront individual situations [80]. Specifically, everyone's psychosocial variables will be assessed, and after the first two weeks, a personalized report with specific recommendations will be provided, to be deliberated upon with each participant in a reflection session focusing on areas that could be prioritized to enhance Self-Efficacy and Health Related Quality of Life.

Supplementing this meaningful learning model as the central framework of the strategy, we have conceived the design of the strategy by progressing the learning objectives in line with modified Bloom's taxonomy [81–83], prioritizing the learning of lower-order thoughts at the beginning, thereby facilitating the application of newly acquired knowledge to manage each patient's pain.

At the end of each session, reinforcement content will be provided in written format, enabling patients to review and start the subsequent session by addressing uncertainties and clarifying previously insufficiently explained queries. These presented contents will be linked to the activities planned for the following day, motivating, and urging patients to not merely wait for in-person sessions, but to proactively engage in their learning process [84]. Moreover, activities designed to facilitate learning in personal spaces will be integrated into the sessions, such as the keeping of pain reflection journals or completion of specific knowledge questionnaires.

In this context, the flipped classroom model [85] will be adapted for this intervention. Instead of assigning completely unfamiliar content for individual learning, as is often the case, priority is given to content that provides a bridge between what has been covered and what will be covered in the future. This approach aims to reduce uncertainty and facilitate the meaningful learning of new complex concepts. It also allows for a more flexible learning model for patients with the neurocognitive changes that are often associated with chronic pain [86].

The introduction of new content will be executed progressively, with reminders of previously introduced content to establish the knowledge base upon which new knowledge can be built. We will also consider the retention rates of new knowledge as outlined by Ebbinghaus in 1885 [87], acknowledging the diminished attention spans commonly observed in patients with chronic pain [88]. It is therefore appropriate to use the biological basis of learning consolidation times, long-term potentiation and the theories proposed by Wozniak (1994) [89] on optimal times for spaced repetition, by controlling sleep performance between sessions as an indicator, to allow sufficient time for consolidation of learning [90].

Furthermore, the selection of objectives for the educational strategy will be informed by elements highlighted in the motivational interviewing process. We will conduct specific actions related to the four processes outlined by Miller and Rollnick in 1991 [91], following adaptations proposed for inclusion in Pain Neuroscience Education by Nijs in 2020 [92]. Other biobehavioral strategies will be incorporated, including individualized therapeutic contracts [93], which have been shown to improve therapeutic alliance and adherence to treatment in some cases. Other interventions may include the development of active coping strategies and the practice of movement representation, to facilitate the integration of new motor patterns used during the intervention [94].

In summary, the future implementation of the POBTE model in this study will provide a holistic approach that not only improves patient's understanding of the physiological mechanisms of pain, but also aims to instigate meaningful changes in pain-related behaviors. By incorporating strategies from a range of health-related disciplines, the model will encourage the development of new behaviors and improve decision-making skills.

This intervention framework will place a strong emphasis on individualized, patient-centered learning, drawing on techniques from classic Ausbel's (1963) [76] meaningful learning model to the most recent flipped classroom model [85]. These methods will optimize the learning process and encourage patients to not only reconceptualize their understanding of pain, but also change their pain related behaviors. For that reason, the framework will incorporate biobehavioral strategies such as individualized therapeutic contracts, active coping strategies, as well as movement imagery techniques to stimulate this desired behavioral change.
