# Supplementary material for: Can a specific biobehavioral-based therapeutic education program lead to changes in pain perception and brain plasticity biomarkers in chronic pain patients? A study protocol for a randomized clinical trial
Source: PLoS One. 2024 Jan 19;19(1):e0289430. doi: 10.1371/journal.pone.0289430 (PMC10798500; doi:10.1371/journal.pone.0289430)
Supplement: S2 File — (DOCX) [file pone.0289430.s006.docx]

*File 6. Informed Consent in Spanish*

**HOJA DE INFORMACIÓN Y CONSENTIMIENTO INFORMADO PARA PARTICIPANTES**

En primer lugar, agradecemos su interés en el presente estudio titulado: *“Efecto de la educación terapéutica sobre la intensidad de dolor y los niveles de BDNF en pacientes con dolor crónico”.*

Gracias por su atención.

*Contacto por si surgen dudas*:

El investigador principal es Silvia Di Bonaventura. Departamento de Fisioterapia, Terapia Ocupacional, Rehabilitación y Medicina Física de la Universidad Rey Juan Carlos - Campus de Alcorcón (Madrid). Teléfono: 655412476. Correo electrónico: [Silvia.dibonaventura@urjc.es](mailto:Silvia.dibonaventura@urjc.es)

**1. ¿Qué es y qué persigue este estudio?**

La educación terapéutica es un método de intervención mediante el cual se pretende dotar al paciente de estrategias de afrontamiento activas ante su dolor. Se ha observado que es una herramienta potente en pacientes con determinados cuadros de dolor crónico siempre que se acompañe de un enfoque activo por parte del paciente y prescrito por el profesional sanitario como puede ser la combinación con el ejercicio terapéutico como en el presente estudio. La combinación de ambas terapias podría favorecer la reversibilidad de los cambios generados en el sistema nervioso central en pacientes con dolor crónico, acelerando la recuperación y proporcionando mejores resultados a largo plazo. Por ello con este estudio pretendemos evaluar los efectos de la educación en los niveles de dolor, frente a un protocolo de solo ejercicio. Como objetivo secundario pretendemos analizar los efectos que se puedan generar sobre los niveles de una proteína cerebral llamada BDNF y diferentes variables psicosociales.

**2. ¿Cómo se realizará el estudio?**

El estudio se realizará en la Clínica Universitaria de la Universidad Rey Juan Carlos (Alcorcón, Madrid). Todo el procedimiento será **totalmente gratuito**. En primer lugar, se solicitará que cumplan los criterios de inclusión del estudio y no presentar ninguno de los criterios de exclusión que se expondrán a continuación, y que podrían poner en riesgo la integridad del estudio y su salud:

**Criterios de inclusión:**

- género masculino y femenino de 18 a 65 años con dolor musculoesquelético desde hace un mínimo de 3 meses,
- no haber recibido tratamiento fisioterápico por este mismo proceso en los últimos 3 meses.
- capacidad para realizar todas las pruebas clínicas y entender el proceso del estudio, así como la obtención del consentimiento informado.

**Criterios de exclusión:**

- enfermedades sistémicas,
- enfermedades neurológicas,
- procesos oncológicos
- procesos inflamatorios,
- patologías psiquiátricas,
- embarazo,
- diabetes tipo II.

Una vez firmado el consentimiento informado, deberá rellenar unos cuestionarios para la medición de *variables sociodemográficas, ansiedad, depresión, catastrofismo, Escala de Gradación del Dolor Crónico, Escala EVA, Cuestionario de conocimientos sobre aspectos específicos del dolor*. Estos cuestionarios nos ayudarán a tener en cuenta factores externos a la intervención que puedan afectar a los resultados de la misma. La estimación del tiempo que se empleará en rellenar dichos cuestionarios es de 20-25 minutos.

Posteriormente se llevará a cabo la evaluación de otras variables por parte de un fisioterapeuta previamente formado en la medición de las mismas, entre las que se encuentran: *Tensión Arterial, frecuencia respiratoria, Saturación y Frecuencia Cardiaca****.*** Una vez completadas estas mediciones, se pasará a la extracción de sangre realizada por una enfermera.

El proceso de evaluación de las variables requerirá de 30 minutos, al haber una evaluación previa a la intervención y otra posterior a la misma el total de las dos evaluaciones será de 1 hora. El investigador le explicará detenidamente en qué consiste cada prueba para que pueda colaborar adecuadamente. Debe conocer que puede decidir cesar la evaluación cuando desee. Tras esta toma de datos inicial será asignado aleatoriamente a uno de los 2 grupos de tratamiento en los que recibirá sesiones de educación y ejercicios salvo en el grupo control activo en el que solo participará en las sesiones de ejercicio terapéutico en días alternos que le será explicado por el personal investigador.

*Grupo intervención (Ejercicio + Educación en Dolor):*

Los pacientes asignados al grupo de intervención serán instruidos por un fisioterapeuta en la realización de las sesiones de ejercicio terapéutico en función de su capacidad funcional, tomando como referencia su FCMax. Se le pautarán las medidas de intensidad de referencia para el incremento esperado en el tiempo de entrenamiento y las indicaciones sobre los riesgos potenciales, y referencias para la interrupción del ejercicio en su caso. La duración total del programa de ejercicio será de 4 semanas, con 3 sesiones de un máximo de 1h por sesión. En los días alternos, 2 veces por semana el paciente recibirá sesiones de Educación en Neurofisiología del Dolor. Su duración será de un máximo de 45 minutos, dos veces por semana durante 4 semanas.

*Grupo control activo (Ejercicio):*

Los pacientes asignados al grupo control activo serán instruidos por un fisioterapeuta en la realización de las sesiones de ejercicio terapéutico en función de su capacidad funcional, tomando como referencia su FCMáx. Se le pautarán las medidas de intensidad de referencia para el incremento esperado en el tiempo de entrenamiento y las indicaciones sobre los riesgos potenciales, y referencias para la interrupción del ejercicio en su caso. La duración total del programa de ejercicio será de 4 semanas, con 2 sesiones de un máximo de 45 minutos por sesión.

La educación en dolor será realizada por un fisioterapeuta con 8 años de experiencia y con formación en la aplicación de dicha herramienta terapéutica. La parte de ejercicio la llevará a cabo otra fisioterapeuta con 12 años de experiencia. Al finalizar las sesiones se solicitará que cumplimente una hoja de efectos adversos que haya podido experimentar durante la intervención. El proceso de evaluación de las variables analizadas volverá a ser realizado tras la primera intervención, a las 2 semanas, a las 4 semanas y a las 4 y 32 semanas tras la finalización de la intervención.

El tiempo total estimado de cada sesión de valoración e intervención será de 1 hora y 45 minutos aproximadamente, mientras que las sesiones de intervención durarán 45-60 minutos aproximadamente.

*Beneficios y riesgos derivados de la intervención*

Con su participación en este estudio estará contribuyendo al desarrollo de la Ciencia y, específicamente a la Fisioterapia. Los efectos adversos más frecuentes (aun siendo muy poco frecuentes) derivados del tratamiento son casi nulos en cuanto a las sesiones de educación en dolor y mínimos en la parte de ejercicio terapéutico. Por otro lado, hay que tener en cuenta que, durante la extracción de sangre, al insertar la aguja, algunas personas sienten un dolor, mientras que otras sólo sienten un pinchazo o sensación de picadura.

**3. ¿Cómo trataremos sus datos?**

Los datos personales que se solicitan están limitados al consentimiento y se utilizará un código anonimizado. Se llevará a cabo la anonimización de datos mediante la asignación de un código numérico y se custodiarán los datos personales bajo llave en el despacho de Silvia Di Bonaventura.

El plazo durante de conservación de los datos personales será de 2 años desde el comienzo del estudio, tiempo necesario para poder reclutar a los pacientes y proceder al análisis estadístico de los datos para la difusión posterior del estudio. El paciente tiene derecho a presentar una reclamación ante una autoridad de control (Reglamento UE 2016/679 artículo 77). El paciente se quedará con una copia firmada de este Consentimiento.

Sepa que la **finalidad del tratamiento** de los datos personales que se deriven de su participación será utilizada únicamente para la investigación. No obstante, le informamos que está contemplado en el Reglamento europeo general de Protección de Datos, Reglamento (UE) 2016/679 ( art. 13.3) que si se diera el caso de que el/la investigador/a responsable del estudio quisiera proyectar el tratamiento ulterior de datos personales para un fin que no sea aquel para el que se recogieron, deberá proporcionarle a usted con anterioridad a dicho tratamiento ulterior la información sobre ese otro fin y todo lo relativo a la información necesaria sobre sus derechos y el tratamiento de los datos.

Usted puede ejercer los derechos de acceso, rectificación, supresión (derecho al olvido), limitación del tratamiento, a la portabilidad de datos, oposición (derecho a la exclusión voluntaria) y derecho a no someterse a la toma de decisiones automatizadas, incluyendo la elaboración de perfiles, poniéndose en contacto con el investigador principal del estudio (abajo firmante). Así mismo tiene derecho a dirigirse a la Agencia de Protección de Datos si no quedara satisfecho.

De igual forma, tiene el derecho de revocar su participación en el estudio en cualquier momento sin tener que dar ninguna explicación ni razón para ejercerlo”.

**4. Sus derechos en materia de protección de datos**

A continuación, en cumplimiento de las obligaciones establecidas en la Ley Orgánica 3/2018, de 5 de diciembre, de Protección de Datos Personales y Garantía de los Derechos Digitales se le facilita la información relativa al tratamiento de sus datos personales:

-Dado que usted está leyendo esta hoja de información, ya que se solicita su participación en un proyecto de investigación, sepa que tiene derecho a recibir previamente toda la información necesaria debidamente documentada y en forma comprensible y mediante los medios adecuados según las necesidades de adaptación que usted requiera para ello. Si no entiende algo no dude en decirlo y en pedir todas las explicaciones que necesite.

- Sepa que sólo tendrán acceso a sus datos los miembros del equipo de investigación, siendo el/la [**responsable último del tratamiento**](https://www.urjc.es/proteccion-de-datos/1035-conceptos#responsable-del-tratamiento-o-responsable) de los datos el/la Investigador Principal. Será con esta persona con la que deberá contactar en la dirección de correo arriba indicada en caso de querer ejercer los derechos que le corresponden en materia de protección de datos.

- Ponemos en su conocimiento que, en cumplimiento del Reglamento europeo general de Protección de Datos, la Universidad Rey Juan Carlos ha designado a una persona como **delegada de protección de datos**, cuyas funciones son de asesoramiento, control y supervisión de los procedimientos y de aplicación de la normativa, así como las relaciones con la Agencia Española de Protección de Datos como autoridad de control y con las personas interesadas. A tal efecto, sepa que podrá contactar con ella en la siguiente dirección de correo: [protecciondedatos@urjc.es](mailto:protecciondedatos@urjc.es).

- Según los **artículos 15 a 22 del Reglamento Europeo (UE) 2016/679** usted tiene derecho a solicitar al responsable del tratamiento de los datos, es decir, al Investigador principal, el acceso a sus datos personales, a su rectificación, a su supresión, a la limitación de su tratamiento, o a oponerse al tratamiento, así como el derecho a la portabilidad de los datos. Sepa, además, que tales derechos podrán ejercerse directamente o por medio de representante legal o voluntario.

- Usted tiene **derecho a retirar/revocar su consentimiento** en cualquier momento, sin que ello afecte a la licitud del tratamiento basado en el consentimiento previo a su retirada o sin que ello le reporte ningún tipo de consecuencia.

-Según el Reglamento UE 2016/679 en su artículo 77, usted puede ejercer su **derecho a presentar una reclamación** ante una [autoridad de control](https://www.aepd.es/es/derechos-y-deberes/conoce-tus-derechos).

**Aquí termina la hoja de información para que piense si acepta participar en el estudio.**

**Gracias por tomarse su tiempo en leerla; como ya le hemos contado debemos respetar la normativa sobre protección de datos y todo lo que ha leído era necesario para que conociera sus derechos.**

**ES EL MOMENTO DE QUE TERMINE DE PREGUNTAR LO QUE NECESITE O NO LE HAYA QUEDADO CLARO, AÚN.**

**Una vez todo aclarado, si decide participar, hay que rellenar y firmar la hoja siguiente, de “Consentimiento informado”, significando que acepta y consiente participar en el estudio después de haber recibido toda la información.**

**CONSENTIMIENTO INFORMADO**

**Yo (nombre del/la participante/paciente**):

Y, reconociendo haber tenido en cuenta sus deseos u objeciones previamente expresados al respecto de este estudio,

confirmo que con esta hoja de consentimiento, me ha sido entregada una hoja de información sobre el estudio que lleva por título:

“**Efecto de la educación terapéutica sobre la intensidad de dolor y los niveles de BDNF en pacientes con dolor crónico”**

Afirmo que he comprendido lo que pone en ella, que se me ha informado sobre mis derechos en materia de protección de datos y se me ha dado la oportunidad de realizar las preguntas que he considerado necesarias para poder entenderlo bien, por lo que manifiesto mi voluntad libre e informada de aceptar voluntariamente mi participación en el estudio; suscribo que me es entregada copia de este consentimiento y consiento de forma expresa, mediante mi firma, el tratamiento de mis datos personales para los fines anteriormente mencionados, en relación con la gestión y ejecución del proyecto de investigación.

En ___________________ a ____ de _______de 20

**Nombre y apellidos del/la participante/ representante: Nombre y apellidos del/la investigador/a:**

**Firma Firma**

**DERECHO DE REVOCACIÓN**

**(En caso de querer ejercer su derecho de retirar su consentimiento)**

**Yo (nombre del/la participante/paciente**)

Y, reconociendo haber tenido en cuenta sus deseos u objeciones previamente expresados al respecto de este estudio.

Revoco el consentimiento informado otorgado previamente a día de hoy …… de ………… de ……….. y no deseo continuar en el estudio que lleva por título: _____________________________________________________________________________________ dándolo por finalizado a partir de la fecha anteriormente descrita. Además, suscribo que me es entregada copia de esta revocación.

**Nombre y apellidos del/la participante/ representante: Nombre y apellidos del/la investigador/a:**

**Firma Firma**

*Contacto por si surgen dudas*:

El investigador principal es Silvia Di Bonaventura. Departamento de Fisioterapia, Terapia Ocupacional, Rehabilitación y Medicina Física de la Universidad Rey Juan Carlos - Campus de Alcorcón (Madrid). Teléfono: 655412476. Correo electrónico: Silvia.dibonaventura@urjc.es
