# Supplementary material for: Can a specific biobehavioral-based therapeutic education program lead to changes in pain perception and brain plasticity biomarkers in chronic pain patients? A study protocol for a randomized clinical trial
Source: PLoS One. 2024 Jan 19;19(1):e0289430. doi: 10.1371/journal.pone.0289430 (PMC10798500; doi:10.1371/journal.pone.0289430)
Supplement: S3 File — (DOCX) [file pone.0289430.s007.docx]

*File 6. Informed Consent in English*

**INFORMATION AND INFORMED CONSENT FORM FOR PARTICIPANTS**

First, we thank you for your interest in the present study entitled: "*Effect of therapeutic education on pain intensity and BDNF levels in patients with chronic pain*". Thank you for your attention.

*Contact in case of questions:*

The principal investigator is Silvia Di Bonaventura. Department of Physiotherapy, Occupational Therapy, Rehabilitation and Physical Medicine, Universidad Rey Juan Carlos - Campus de Alcorcón (Madrid). Telephone: 655412476. E-mail: [Silvia.dibonaventura@urjc.es](mailto:Silvia.dibonaventura@urjc.es)

**1. What is it and what is the purpose of this study?**

Therapeutic education is a method of intervention that aims to provide patients with active coping strategies to deal with their pain. It has been observed that it is a powerful tool in patients with certain chronic pain conditions, provided that it is accompanied by an active approach on the part of the patient and prescribed by the health professional, such as the combination with therapeutic exercise, as in the present study. The combination of both therapies could favor the reversibility of the changes generated in the central nervous system in patients with chronic pain, accelerating recovery and providing better long-term results. Therefore, with this study we intend to evaluate the effects of education on pain levels, compared to an exercise-only protocol. As a secondary objective we intend to analyze the effects that can be generated on the levels of a brain protein called BDNF and different psychosocial variables.

**2. How will the study be conducted?**

The study will be performed at the “Clínica Universitaria de la Universidad Rey Juan Carlos” (Alcorcón, Madrid). The entire procedure will be **completely free of charge**. First of all, you will be asked to meet the inclusion criteria of the study and not to present any of the exclusion criteria that will be exposed below, and that could jeopardize the integrity of the study and your health:

**Inclusion criteria:**

- male and female, aged 18 to 65 years with musculoskeletal pain for at least 3 months,

- not having received physiotherapy treatment for this same process in the last 3 months.

- Ability to perform all clinical tests and to understand the study process, as well as to obtain informed consent. 
 

**Exclusion criteria:**

- systemic diseases,

- neurological diseases,

- oncological processes

- inflammatory processes,

- psychiatric pathologies,

- pregnancy,

- type II diabetes.

Once the informed consent has been signed, the patient should fill in questionnaires for the measurement of sociodemographic variables, anxiety, depression, catastrophism, sleep quality, quality of life, Chronic Pain Grading Scale, EVA Scale, physical activity levels, Pressure Pain Threshold, Questionnaire of knowledge about specific aspects of pain. These questionnaires will help us to take into account factors external to the intervention that may affect the results of the intervention. The estimated time to fill in these questionnaires is 20-25 minutes.

Subsequently, the evaluation of other variables will be carried out by a physiotherapist previously trained in the measurement of the same, among which are: Blood Pressure, Respiratory Rate, Saturation and Heart Rate. Once these measurements have been completed, blood will be drawn by a nurse.

The evaluation process of the variables will require 30 minutes, since there will be a pre-intervention evaluation and a post-intervention evaluation, the total of the two evaluations will be 1 hour.  The researcher will explain carefully what each test consists of so that you can collaborate adequately. You should be aware that you can decide to stop the assessment whenever you wish. After this initial data collection, you will be randomly assigned to one of the 2 treatment groups in which you will receive education and exercise sessions, except in the active control group in which you will only participate in the therapeutic exercise sessions on alternate days, which will be explained to you by the researcher.

*Intervention group (Exercise + Pain Education):*

The patients assigned to the intervention group will be instructed by a physiotherapist in the performance of the therapeutic exercise sessions according to their functional capacity, taking their FCMax as a reference. The reference intensity measures for the expected increase in training time and indications about potential risks and references for the interruption of exercise, if necessary, will be given. The total duration of the exercise program will be 4 weeks, with 3 sessions of a maximum of 1h per session. On alternate days, 2 times per week the patient will receive sessions of Pain Neurophysiology Education. Its duration will be a maximum of 45 minutes, twice a week for 4 weeks.

*Active control group (Exercise):*

The patients assigned to the active control group will be instructed by a physiotherapist in the performance of the therapeutic exercise sessions according to their functional capacity, taking as a reference their HRmax. They will be given the reference intensity measures for the expected increase in training time and indications on the potential risks, and references for the interruption of the exercise if necessary. The total duration of the exercise program will be 4 weeks, with 2 sessions of a maximum of 45 minutes per session.

The pain education will be performed by a physiotherapist with 8 years of experience and training in the application of this therapeutic tool. The exercise part will be carried out by another physiotherapist with 12 years of experience. At the end of the sessions, the patient will be asked to fill out a sheet of adverse effects that may have been experienced during the intervention. The evaluation process of the variables analyzed will be carried out again after the first intervention, at 2 weeks, at 4 weeks and 8 weeks after the end of the intervention.

The total estimated time for each assessment and intervention session will be approximately 1 hour and 45 minutes, while the intervention sessions will last approximately 45-60 minutes.

*Benefits and risks derived from the intervention*

With your participation in this study you will be contributing to the development of Science and, specifically to Physiotherapy. The most frequent adverse effects (although very infrequent) derived from the treatment are almost nil in the pain education sessions and minimal in the therapeutic exercise part. On the other hand, it should be taken into account that, during blood collection, when inserting the needle, some people feel a pain, while others only feel a prick or stinging sensation.

**3. How will we handle your data?**

The personal data requested is limited to consent and an anonymized code will be used. Data anonymization will be carried out by assigning a numerical code and the personal data will be kept under lock and key in Silvia Di Bonaventura's office.

The period of storage of personal data will be 2 years from the start of the study, which is necessary to be able to recruit patients and proceed with the statistical analysis of the data for the subsequent dissemination of the study. The patient has the right to lodge a complaint to a supervisory authority (EU Regulation 2016/679 Article 77). The patient will keep a signed copy of this Consent.

Please note that the purpose of the processing of personal data arising from your participation will be used only for research purposes. However, we inform you that it is provided for in the General European Data Protection Regulation, Regulation (EU) 2016/679 ( art. 13.3) that if it should be the case that the researcher responsible for the study would like to plan the further processing of personal data for a purpose other than that for which it was collected, he/she must provide you prior to such further processing with information about that other purpose and everything related to the necessary information about your rights and the processing of the data.

You may exercise your rights of access, rectification, erasure (right to be forgotten), limitation of processing, data portability, objection (right to opt-out) and the right not to be subject to automated decision-making, including profiling, by contacting the principal investigator of the study (undersigned). You also have the right to contact the Data Protection Agency if you are not satisfied.

Similarly, you have the right to revoke your participation in the study at any time without having to give any explanation or reason for exercising it."

**4. Your data protection rights**

Below, in compliance with the obligations established in the Organic Law 3/2018, of December 5, on the Protection of Personal Data and Guarantee of Digital Rights, you are provided with the information regarding the processing of your personal data:

-Since you are reading this information sheet, as you are being asked to participate in a research project, please know that you have the right to receive in advance all the necessary information duly documented and in an understandable form and through the appropriate means according to the adaptation needs you require for it. If you do not understand something, do not hesitate to say so and ask for all the explanations you need.

-Please note that only members of the research team will have access to your data, the Principal Investigator being ultimately responsible for data processing. This is the person you should contact at the e-mail address above if you wish to exercise your data protection rights.

-We inform you that, in compliance with the General European Data Protection Regulation, the Universidad Rey Juan Carlos has designated a person as data protection officer, whose functions are to advise, control and supervise the procedures and application of the regulations, as well as relations with the Spanish Data Protection Agency as supervisory authority and with the persons concerned. For this purpose, you may contact her at the following e-mail address: [protecciondedatos@urjc.es](mailto:protecciondedatos@urjc.es).

-According to **Articles 15 to 22 of the European Regulation (EU) 2016/679** you have the right to request from the data controller, i.e. the Principal Investigator, access to your personal data, rectification, erasure, restriction of processing, or to object to processing, as well as the right to data portability. Please note that these rights may be exercised directly or through a legal representative or volunteer.

-You have the right to withdraw/revoke your consent at any time, without affecting the lawfulness of the processing based on the consent prior to its withdrawal or without any consequences for you.

- According to EU Regulation 2016/679 in Article 77, you may exercise your right to lodge a complaint with a [control authority](https://www.aepd.es/es/derechos-y-deberes/conoce-tus-derechos).

**THIS IS THE END OF THE INFORMATION SHEET FOR YOU TO CONSIDER WHETHER YOU AGREE TO PARTICIPATE IN THE STUDY.**

Thank you for taking the time to read it; as we have already told you, we must respect data protection regulations and everything you have read was necessary for you to know your rights.

**NOW IT IS TIME FOR YOU TO FINISH ASKING ANY QUESTIONS YOU NEED TO ASK OR HAVE NOT YET CLARIFIED.**

Once everything has been clarified, if you decide to participate, you must fill in and sign the following sheet, "informed consent", meaning that you accept and consent to participate in the study after having received all the information. 

**INFORMED CONSENT**

**I  (name of participant/patient**):

And acknowledging having taken into account their previously expressed wishes or objections with respect to this study,

I confirm that with this consent form, I have been provided with an information sheet about the study entitled:

“**Effect of therapeutic education on pain intensity and BDNF levels in patients with chronic pain.”**

I affirm that I have understood what it says in it, that I have been informed about my rights regarding data protection and have been given the opportunity to ask the questions I have considered necessary to understand it well, so I express my free and informed will to voluntarily accept my participation in the study; I subscribe that I am given a copy of this consent and I expressly consent, by my signature, the processing of my personal data for the purposes mentioned above, in connection with the management and execution of the research project.

In ___________________  ____ of _______of 20

**Name and surname of the participant/ representative:                       Name and surname of the researcher:** 
 

**Signature                                                                                                      Signature**

 
 

**RIGHT OF REVOCATION**

**(In case you wish to exercise your right to withdraw your consent)**

**I (name of participant/patient**)

And acknowledging having considered their previously expressed wishes or objections with respect to this study.

I revoke the informed consent previously given as of today ............ from ........... and I do not wish to continue in the study entitled: _____________________________________________________________________________________

I further agree to terminate this agreement as of the date described above. I further subscribe that a copy of this revocation is delivered to me.

**Name and surname of the participant/ representative:                             Name and surname of the researcher:**

**Signature**  **Signature**

*Contact in case of questions*:

The principal investigator is Silvia Di Bonaventura. Department of Physiotherapy, Occupational Therapy, Rehabilitation and Physical Medicine, Universidad Rey Juan Carlos - Campus de Alcorcón (Madrid). Telephone: 655412476. E-mail: [Silvia.dibonaventura@urjc.es](mailto:Silvia.dibonaventura@urjc.es)
