# Supplementary material for: Can a specific biobehavioral-based therapeutic education program lead to changes in pain perception and brain plasticity biomarkers in chronic pain patients? A study protocol for a randomized clinical trial
Source: PLoS One. 2024 Jan 19;19(1):e0289430. doi: 10.1371/journal.pone.0289430 (PMC10798500; doi:10.1371/journal.pone.0289430)
Supplement: Attachment — Submitted filename: renamed_58d3c.docx [file pone.0289430.s008.docx]

# COMMENTS – Manuscript PONE-D-23-22066

Title: “Can a specific biobehavioral based therapeutic education program lead to changes in pain perception and brain plasticity biomarkers in chronic pain patients? A study protocol for a randomized clinical trial.”

**Important note: This review pertains only to ‘statistical aspects’ of the study and so ‘clinical aspects’ [like medical importance, relevance of the study, ‘clinical significance and implication(s)’ of the whole study, etc.] are to be evaluated [should be assessed] separately/independently. Further please note that any ‘statistical review’ is generally done under the assumption that (such) study specific methodological [as well as execution] issues are perfectly taken care of by the investigator(s). This review is not an exception to that and so does not cover clinical aspects {however, seldom comments are made only if those issues are intimately / scientifically related & intermingle with ‘statistical aspects’ of the study}. Agreed that ‘statistical methods’ are used as just tools here, however, they are vital part of methodology [and so should be given due importance]. I look at the manuscript in/with statistical view point, other reviewer(s) look(s) at it with different angle so that in totality the review is very comprehensive. However, there should be efforts from authors side to improve (may be by taking clues from reviewer’s comments). Therefore, please do not limit the revision only (with respect) to comments made here.**

**COMMENTS:** I note that this manuscript is well drafted [and the study protocol is alright with respect to few aspects], I have certain definite observations/concerns (different opinion) which are given below:

Firstly, the account given in section ‘Sample Size’ (lines 354-368) appears alright, however, mind you that the effect size of 0.94 assumed is very large [your calculations are correct but the assumption regarding effect size in not at all reasonable, in my opinion]. Whereas details given of “POBTE’s intervention” are appreciable, difference from “Active Control group (Exercise)” [as indicated/implied by effect size assumed] cannot be that large [generally for such studies on non-pharmacological interventions], I guess. Effect size assumed here while estimating required sample size is very large [most likely], by any standard and please note that the ‘effect size’ assumed should be reasonable/realistic, else the study is very likely ‘not to be able to’ detect a difference despite its presence. In this case ‘effect size’ can at best be ‘medium’. The quoted reference [56] by Cohen J. ‘Statistical power analysis for the behavioral sciences’ is excellent, however, it not properly used {used to estimate SDpooled, but data used were from some pilot study, as this reference [Cohen’s] never comment(s) on likely ‘effect size’}.

According to table-2 on page 158 of Jacob Cohen’s paper “A power primer” in Psychological Bulletin, 1992, vol.:112, pp 155-159 [which is a sort of summary of the above quoted excellent book by Cohen himself for medium effect size you need n=64 per group (type-I error=0.05, power=80%). Your answer is [lines 364-5] “resulting in the need for at least 25 subjects per group to be able to detect a 2-point difference in VAS with 90% power” and in line 367, you said “Consequently, the study will require a total of 64 subjects to reach the required statistical power”. Note that my question is not about software used [GPower or reference 57] as this software is excellent. For the same assumption of drop-out rate {25% is in fact, which is much more than generally allowed in any standard clinical trial(s)}, the study will require a total of 160 subjects to reach even 80% power.

In the context of ‘**Statistics: additional analyses**’ section (lines 430 onwards), mind you that ‘Multiple linear regression models (this technique [in fact any regression technique(s) for that matter] is/are not originally developed for testing the ‘Group difference(s)’. Head-to-head comparison is expected, as this is an indirect/secondary/by-product testing, in my opinion). You said [lines 432-33] “Subgroup analyses will consider age, gender, and duration of pain” is very good but do you think that this sample size will suffice to do this? Moreover, note that though the measures/tools used are appropriate [examples: *Pain intensity - Visual Analogue Scale (VAS); Plasma BDNF levels;* HADS self-completion scale; EuroQoL-5D questionnaire (EQ-5D); Pain Catastrophism Scale (ECD); *Chronic Pain Grading Scale (CPGS)*], most of them are likely to yield data that are in ‘ordinal’ level of measurement [and not in ratio level of measurement for sure {as the score two times higher does not indicate presence of that parameter/phenomenon as double}]. Then application of suitable non-parametric (or distribution free) test(s) is/are indicated/advisable [even if distribution may be ‘Gaussian’ (also called ‘normal’)]. Agreed that there is/are no non-parametric test(s)/technique(s) available to be used as alternative in all situation(s), but should be used whenever/wherever they are available. Therefore, in short use suitable non-parametric test(s)/technique(s) while dealing with data that are in ‘ordinal’ level of measurement even if [despite that] the distribution may be ‘Gaussian’. Testing ‘normality’ in sample [by using any normality test(s)} is not required/desired while dealing with data that are in ‘ordinal’ level of measurement [as most of the normality tests are not valid for ‘ordinal’ data].

In ‘Conclusion’ section of abstract [lines 24-25] you conclude that “The rigorous scientific methods used ensure that the proposed interventions will be clinically applicable across different health care systems” is not understood. What type of conclusion it is? How this study helped the authors to conclude this? In any case, remember/mind you that this is a scientific/academic document and so all details should be clearly/correctly communicated (do not take readers’ for granted).

Refer to line 379 [stratified permuted block (block size of 4 and 6)], Please explain the role of ‘stratification’ in ‘permuted block’ randomization’? {i.e. how you used/executed in this study?} Moreover, please note that while using ‘permuted block randomization’, we generally do not reveal ‘block size’ [which is randomly chosen]. Further, please refer to lines 425-27 [ANOVA test will be used to analyze the group factor in the quantitative variables and if the ANOVA reveals statistical significance, a post-hoc analysis with Bonferroni correction will be performed], according to my understanding, you may not need ANOVA and subsequent any post-hoc analysis as there are only two groups.

In the beginning of the article (line 29) there is a list ‘**Strengths and limitations of this study**’ where there is hardly any limitation(s). Does that mean {according to authors} there are none?

As pointed out in ‘important note’ above “This review pertains only to ‘statistical aspects’ of the study and so ‘clinical aspects’ should be assessed separately/independently [one should carefully consider/look at the clinical implications of the study]. In my opinion, to make this article acceptable (which is not impossible), large amount of re-vision (re-drafting) may be needed. However, please do not limit the revision only (with respect) to comments made here [as this is a protocol and is possible now to accommodate suggestion by other respected reviewers. ‘Major revision’ is recommended.
