# Supplementary material for: Can a specific biobehavioral-based therapeutic education program lead to changes in pain perception and brain plasticity biomarkers in chronic pain patients? A study protocol for a randomized clinical trial
Source: PLoS One. 2024 Jan 19;19(1):e0289430. doi: 10.1371/journal.pone.0289430 (PMC10798500; doi:10.1371/journal.pone.0289430)
Supplement: Attachment — Submitted filename: ReviewerComments.docx [file pone.0289430.s010.docx]

**Responses to Reviewer’ comments:**

*Dear reviewer, thank you again for your comments.*

***REVIEWER***

*1.- In response to* “Although all the comments are answered and few positively attended, frankly speaking I do not agree with Rhemtulla et al. (Psychol Methods, 2012 Sep;17(3):354-73) who suggested that under certain conditions, categorical variables with more than five categories can be treated as continuous [quoted as part of answer to my comment].*”*

*We understand your discrepancy, but we would like to emphasize that the method used in our study, based on the aforementioned study by Rhemtulla et al 2012, has been widely cited and accepted by the scientific community, specifically it has been used in more than 395 articles, including 14 in Q1 journals and 88 in Q2 of the JCR, and has even been used and referenced in previous studies published in prestigious journals, including PLOS ONE, as in the article with PMID 37603578 or the one with PMID 37310969, for example. We consider that such a method is sufficiently validated and explained, and that it is relevant to our study, although we acknowledge that it could influence future results in some way, based on the concerns of reviewer 1. This point has been included in the potential limitations of our study in the manuscript.*

*2.- In response to*:

“Look at few of their statements like “In our initial submission, we incorrectly mentioned 'stratification' in the context of our 'permuted block' randomization. This was a misinterpretation on our part.” Which hopefully throw light on quality of work.”

*We also consider that the methodological quality of our work is well documented and we believe that we have correctly responded to all the considerations set out in detail in the previous review, having considerably improved the initial manuscript, and we believe that our study meets the necessary quality requirements to be considered for publication, we regret if an error we made in the drafting of the first version of the manuscript, as English is not our native language, and which we have corrected and submitted for professional editing, is put forward as an example of the quality of the manuscript, but we can do nothing other than apologize for the error made initially and corrected subsequently. Nevertheless, we sincerely thank the reviewer 1 for the time spent in improving our manuscript.*

*Sincerely,*

*The authors.*
